# A mystery revealed: an update on eosinophil and other blood cell morphology of the Argentine black and white tegu (*Salvator merianae*)

**DOI:** 10.3389/fvets.2024.1387178

**Published:** 2024-06-13

**Authors:** Sarah N. Bosch, Nicole I. Stacy, Anibal G. Armien, Charlotte Hollinger, Rashea Minor, Darryl J. Heard, Tracy Stokol

**Affiliations:** ^1^Department of Comparative, Diagnostic, and Population Medicine, College of Veterinary Medicine, University of Florida, Gainesville, FL, United States; ^2^Department of Large Animal Clinical Sciences, College of Veterinary Medicine, University of Florida, Gainesville, FL, United States; ^3^California Animal Health and Food Safety Laboratory Systems (CAHFS), School of Veterinary Medicine, University of California, Davis, Davis, CA, United States; ^4^Inotiv, Inc., Kalamazoo, MI, United States; ^5^Wildlife Conservation Society, Zoological Health Program, Bronx Zoo, Bronx, NY, United States; ^6^Department of Population Medicine and Diagnostic Sciences, College of Veterinary Medicine, Cornell University, Ithaca, NY, United States

**Keywords:** lizard, Teiidae, inclusion, cytochemistry, hematology, leukocyte morphology, white blood cells, electron microscopy

## Abstract

Reptile white blood cell (WBC) morphological features are strikingly variable across species. In the Argentine black and white tegu (*Salvator merianae*), red tegu (*Salvator rufescens*), and Savannah monitor (Var*anus exanthematicus*), previous reports described a WBC type with a single distinct, clear, linear- to ovoid- to crescent-shaped inclusion of presumptive monocytic origin. The objective of this study was to further investigate the origin of this unique WBC type with crescent-shaped inclusions. Blood samples from two Argentine black and white tegus, tegu 1, a 4-year-old female, and tegu 2, a 2-year-old presumed male, were submitted for routine hematological evaluation. Additional blood films were prepared and stained with these cytochemical stains: alkaline phosphatase (ALP; naphthol AS-MX phosphate substrate), alpha-naphthyl butyrate esterase, alpha-chloroacetate esterase, myeloperoxidase, Periodic acid-Schiff, and Sudan black B. Blood films from tegu 1 were also stained with a second ALP stain (5-bromo-4-chloro-3-indoxyl-phosphate and nitroblue tetrazolium substrate), Luna, luxol fast blue, and toluidine blue. The blood from tegu 1 was cytocentrifuged to isolate and fix the buffy coat in glutaraldehyde 2.5% aqueous solution for transmission electron microscopy. Six morphologically distinct WBC types were identified from tegu 1, including heterophils, basophils, monocytes, azurophils, lymphocytes, and the unique WBC type, which were identified as eosinophils with inclusions. WBC types in tegu 2 were similar; however, eosinophils lacked a discernable inclusion. Proper WBC identification will be useful in obtaining accurate hemogram data for this species.

## Introduction

Hematological evaluation of reptiles can be challenging due to species differences in white blood cell (WBC) morphology, proportions, and cytochemical staining patterns (1–3). Accurate light microscopic differentiation of a given species in health is important for obtaining reference intervals and interpreting leukogram changes, both of which can be affected by various pre-analytical intrinsic and extrinsic factors (e.g., environment, sex, diet, and methodology) (1, 3). Argentine black and white tegus (*Salvator merianae*) are large, omnivorous South American lizards (order Squamata, family Teiidae) closely related to whiptail lizards (genus *Aspidoscelis* and *Cnemidophorus*) (4, 5). They are common in the international live animal trade as pets, are an established invasive species in Florida and Georgia in the United States, and are hunted for their skins in various South American countries (4). Previous publications have reported that eosinophils in the Argentine black and white tegu are indistinguishable from heterophils with the May-Grűnwald-Giemsa stain (6–8). Monocytes in this species are described as containing an electron-lucent variably shaped inclusion (6), while another report describes WBCs containing oval to linear cytoplasmic clearings of unknown origin in an apparently healthy red tegu (*Salvator rufescens*) and a Savannah monitor (Var*anus exanthematicus*) (3), which is from a different taxonomic family (Varanidae). The objective of this study was to perform a suite of cytochemical staining and transmission electron microscopy (TEM) on blood submitted for a complete blood count (CBC) from one Argentine black and white tegu to identify the cellular origin of the WBC with the previously described inclusion/cytoplasmic clearing that has been a mystery to date. These results were compared to a second Argentine black and white tegu, which lacked these inclusions, to further characterize WBCs and erythrocytes and thrombocytes in these reptiles.

## Methods

Blood was collected on presentation from both animals as part of routine hematological testing after being admitted for evaluation of clinical disease. For tegu 1, heparinized blood (lithium heparin microtainer, Sarstedt AG & Co. KG, Nűmbrecht, Germany) was submitted for a CBC. For CBC evaluation, a packed cell volume (PCV) was determined with a microhematocrit centrifuge (Sorvall™ Legend™ Micro 17 Microcentrifuge, Thermo Fisher Scientific, Massachusetts, USA), and total protein (TP) of plasma was assessed by refractometer (Rhino ® Vet 360 refractometer, Ametek Reichert, New York, USA). A WBC count could not be obtained using a Neubauer hemocytometer and Natt and Herrick solution due to abundant leukergy. A WBC was estimated, and a 200 WBC differential count was performed on a Wright-Giemsa-stained (Aerospray Hematology Pro, ELITechGroup Inc., Utah, USA) blood film by one reviewer (SB). The manual WBC estimate was obtained by multiplying the average number of leukocytes in ten 50x objective fields in the monolayer and multiplying that number by the objective (50x) squared (9).

For tegu 2, a CBC was performed on EDTA-anticoagulated whole blood (BD microtainer®, BD Biosciences, Franklin Lakes, NJ). A PCV was determined with a microhematocrit centrifuge (Pico 17 centrifuge, Haraeus, Thermo Fisher Scientific), and TP of plasma was measured using a digital refractometer (Palm Abbe Digital Refractometer, Misco, Cleveland, OH). A WBC count was obtained using a Neubauer hemocytometer and Phloxine B solution (10). The phloxine B solution was made in-house from powder (Sigma-Aldrich, St Louis, MO). The total WBC count was based only on the heterophil percentage due to the light basophilic appearance of the eosinophils and their unknown uptake with the eosin stain. The equation used to determine the total calculated WBC count in this specific case was [(total count of eosin-stained WBCs on both sides of hemocytometer) x 1.1 × 16 × 100] / % heterophils from the differential (10). A 200 WBC differential count was performed on a blood film stained with modified Wright’s stain (Hema-tek 1,000, Siemens Healthcare Diagnostics Inc., Tarrytown, NJ) by one reviewer (TS); the calculated WBC count was concordant with the WBC estimate, as described above, from the blood film.

Additional blood films for both tegus were prepared for cytochemical staining on the same day as slide submission in both tegus. The following staining reactions were done: Alkaline phosphatase (ALP), α-naphthyl butyrate esterase (ANBE), chloroacetate esterase (CAE), Luna, luxol fast blue (LFB), myeloperoxidase (MPx), Periodic acid-Schiff (PAS), Sudan Black B (SBB), and toluidine blue (TB). Two versions of the ALP stain, one using naphthol AS-MX phosphate and Fast blue RR salt (AS-MX ALP) as substrates (Sigma-Aldrich) and the other using 5-bromo-4-chloro-3-indoxyl-phosphate (BCIP) and nitroblue tetrazolium (NBT) as substrates (KPL ALP) (KPL BCIP/NBT 1- Component Phosphatase Substrate kit, SeraCare, Milford, MA) were compared. The KPL ALP, Luna, LFB, and TB were not done in tegu 2. All stains, with the exception of LFB, were performed in the Animal Health Diagnostic Center in the College of Veterinary Medicine at Cornell University. LFB staining was performed at the Diagnostic Laboratories at the University of Florida College of Veterinary Medicine. Commercially available kits (AS-MX ALP, Procedure No. 85; ANBE, Procedure No. 181; CAE, Procedure No. 91; SBB, Procedure No. 380; Sigma-Aldrich) and an in-house assay for MPx (11) were utilized as previously described in mammalian blood (11, 12), with blood from a horse (ALP) and a dog (ANBE, CAE, MPx, and SBB) as positive controls. For the KLP ALP, slides were fixed in a citrate-acetone buffer used for the AS-MX ALP and then incubated in a Coplin jar with 40 mL of substrate for 15 min at room temperature. Slides were rinsed with distilled water for 30 s, then counterstained with Mayer’s hematoxylin (the kit stain counterstain for AS-MX ALP) for 1 min, rinsed with tap water, and allowed to air dry. Luna, LFB, PAS, and TB stains were performed via previously established protocols in the histology laboratory with histological sections of eosinophilic infiltrates, brain, gastrointestinal tract, and a mast cell tumor as positive controls (12–14). Blood films stained with ALP, ANBE, CAE, Luna, MPx, PAS, SBB, and TB were evaluated by one reviewer (TS), while the LFB-stained blood film was evaluated by three reviewers independently (SB, NS, and TS). Grading was similar to that previously described by Kehoe et al. (12) for the Giant panda. The pattern of staining was described as cytoplasm (C) or granular (G) with degree of staining graded as negative (−), equivocal (negative or weakly positive, +/−), and positive (+) with subsequent grading of positive reactions as weak (+), moderate (++), or strong (+++).

For transmission electron microscopy (TEM), a tube of heparinized blood from tegu 1 was centrifuged at 2,500 *g* for 10 min, the plasma was removed, and the remaining buffy coat was preserved by replacing the plasma volume with glutaraldehyde 25% aqueous solution (MP Biomedicals, Solon, OH, USA). The sample was placed vertically in a refrigerator (4°C) for 24 h, undisturbed, and submitted to the California Animal Health & Food Safety Laboratory (Davis, CA) for TEM processing. In brief, the buffy coat was post-fixed in Karnovsky’s fixative (3% glutaraldehyde and 2% formaldehyde in 0.1 M phosphate buffer, pH 7.4). Using gel as a cohesive matrix, WBCs were pelletized. One cubic millimeter pellets were post-fixed with 1% osmium tetroxide in 0.1 M sodium cacodylate buffer. White blood cell pellets were processed as described elsewhere (15). Samples were dehydrated in an ethyl alcohol gradient series using an automated tissue processor (Leica Microsystems, Wetzlar, Germany) and then infiltrated and embedded in EMbed 812 resin, followed by incubation at 58°C for 24 h to polymerize the resin. All reagents were from Electron Microscopy Sciences (Pennsylvania, USA). Resin blocks were sectioned on a Leica UC7 ultramicrotome (Leica Microsystems, Wetzlar, Germany). From the selected areas, thin sections (70–90 nm) were obtained and contrasted with 5% uranyl acetate and Satos’ lead citrate. All samples were visualized using a JEOL 1400Plus transmission electron microscope (JEOL LTD, Tokyo, Japan). Images were obtained and analyzed using a OneView Camera Model 1,095 with the Gatan Microscope Suite 3.0 (Gatan Inc., California, USA). Blood from tegu 2 was not evaluated using TEM.

## Results

Tegu 1, a 4-year-old female Argentine black and white tegu, was presented to the University of Florida for ascending tail necrosis that continued to worsen despite multiple tail amputation surgeries and antimicrobial treatment. A culture of the tail lesion performed by the referring veterinarian yielded *Klebsiella* spp. and *Enterobacter cloacae*. A blood culture was negative, and radiographic findings were compatible with the visible tail necrosis. Since a specific cause for persistent tail necrosis was not revealed, long-term antibiotic treatment was recommended. Tegu 2, a 2-year-old, presumed male, Argentine black and white tegu, was presented to Cornell University Hospital for Animals for chronic respiratory distress and coelomic distention that persisted despite treatment with furosemide. Computed tomography showed marked coelomic effusion and multiple mid to caudal coelomic cystic structures of unknown origin, causing a mass effect on additional organs and resulting in the restriction of pulmonary inflation. Cytological analysis of an aspirated sample of the effusion was compatible with a protein-poor transudate, and the fluid was negative for bacterial culture. The patient was treated with antibiotics and subsequently lost to follow-up.

**Figure 1:**
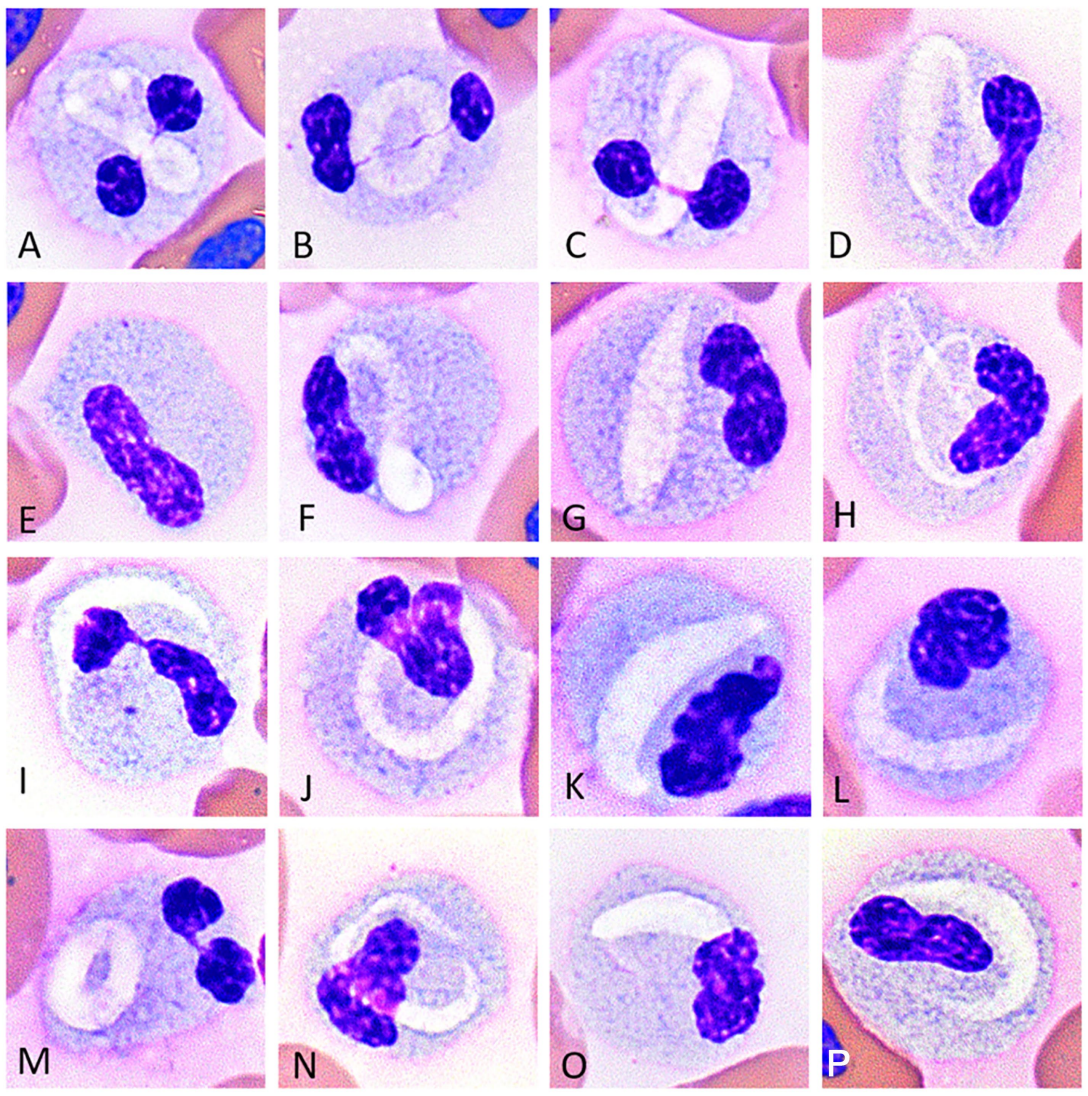
Image composite of unique white blood cells (presumptive eosinophils) of the Argentine black and white tegu (*Salvator merianae*), tegu 1, showing morphological variability in eosinophils with variably sized and shaped pleomorphic pale staining intracellular inclusions **(A-D,F-P)**. Rare cells lacked inclusions; **(E)** Wright-Giemsa-stain x100 objective.

The CBC data are reported in Supplementary Table S1. In tegu 1, six types of WBCs were identified upon blood film review: heterophils, basophils, monocytes, azurophils, lymphocytes, and a unique WBC type with a linear- to crescent-shaped inclusion which has been previously categorized as a monocyte by Chamut et al. (6) (Figures 1A-D,F-P); these cells were initially classified as “other” in the WBC differential and then described in a descriptive comment. In tegu 2, heterophils, eosinophils, basophils, azurophils, and lymphocytes were identified; no cells had crescentic inclusions. The cells classified as eosinophils in tegu 2 resembled the unique WBC with cytoplasmic inclusions in tegu 1 on Romanowsky-based stained smears, were present in similar proportions, and had similar cytochemical staining characteristics (Table 1), supporting that they were a variant of an eosinophil and were reclassified as such in tegu 1.

**Table 1:** Cytochemical staining characteristics of leukocytes and thrombocytes in the Argentine black and white tegu (*Salvator merianae*) with nine different cytochemical stains.

Cell type	Animal ID	AS-MX ALP	KPL ALP	ANBE	CAE	Luna	LFB	MPx	PAS	SBB	TB
Heterophil	Tegu 1	+/−; C: + stippling	C: + diffuse	C: +++	G: ++ to +++	G: ++	G: +++	G: +++ (tegu 1) + (tegu 2)	C: + to ++	G: ++	–
	Tegu 2	–	ND			ND	ND		+/−; C: +		ND
Eosinophil	Tegu 1 with inclusions	–	–	–	G: +++Tegu 1 Inclusions: +++	–	G: +/−	–	C: +	–	–
	Tegu 2	C: + stippling	ND			ND	ND				ND
Basophil	Tegu 1	NI	NI	–	–	–	–	–	−/+; C: +	–	−/+; G: +
	Tegu 2	–	ND			ND	ND		G: ++ to +++		ND
Azurophil	Tegu 1	C: +	C: ++ diffuse	−/+; C: +	G: +	–	−/+; C: +	G: +	C: + to ++	−/+; G: +	–
	Tegu 2	–	ND		G: + to ++ stippling	ND	ND	–			ND
Monocyte	Tegu 1	–	NI	−/+; C: +	G: +	–	NI	G: +	C: + to ++	–	–
Lymphocyte	Tegu 1	–	–	–	+/−; C: + (tegu 2)++ (tegu 1)	–	–	–	–	–	–
	Tegu 2		ND			ND	ND				ND
Thrombocyte	Tegu 1	NI	NI	NI	–	NI	NI	NI	C: + to ++	NI	–
	Tegu 2	–	ND	–		ND	ND	–		–	ND

C, Cytoplasm; G, Granules; ALP, alkaline phosphatase with different substrates (AS-MX ALP, AS-MX/fast blue RR; KPL ALP, BCIP/NT substrate); ANBE, α-naphthyl butyrate esterase; CAE, Chloroacetate esterase; LFB, Luxol fast blue; MPx, Myeloperoxidase; PAS, Periodic Acid-Schiff; SBB, Sudan black B; TB, Toluidine blue; −/+: Indicates positive and negative cells. ND, not done; NI, not conclusively identified. Granule staining for the heterophils of Tegu 1 had edge to diffuse stain uptake for Luna and focal edge stain uptake for LFB. Eosinophils for Tegu 1, when positive, had focal edge stain uptake with LFB.

### Heterophils

Heterophils were similar in size, ~15–17 μm, and had morphological features similar to those described previously using Wright-Giemsa stain and TEM in this species (6). The majority of heterophils had colorless cytoplasm filled with abundant elongate bright eosinophilic granules in blood films stained with the Romanowsky-based stain. Heterophils in tegu 1 rarely exhibited mild toxic change, and a few immature stages were also observed. The nucleus, when visible, was segmented with clumped chromatin in mature heterophils and band-shaped or reniform with lightly clumped chromatin in immature heterophils.

**Figure 2:**
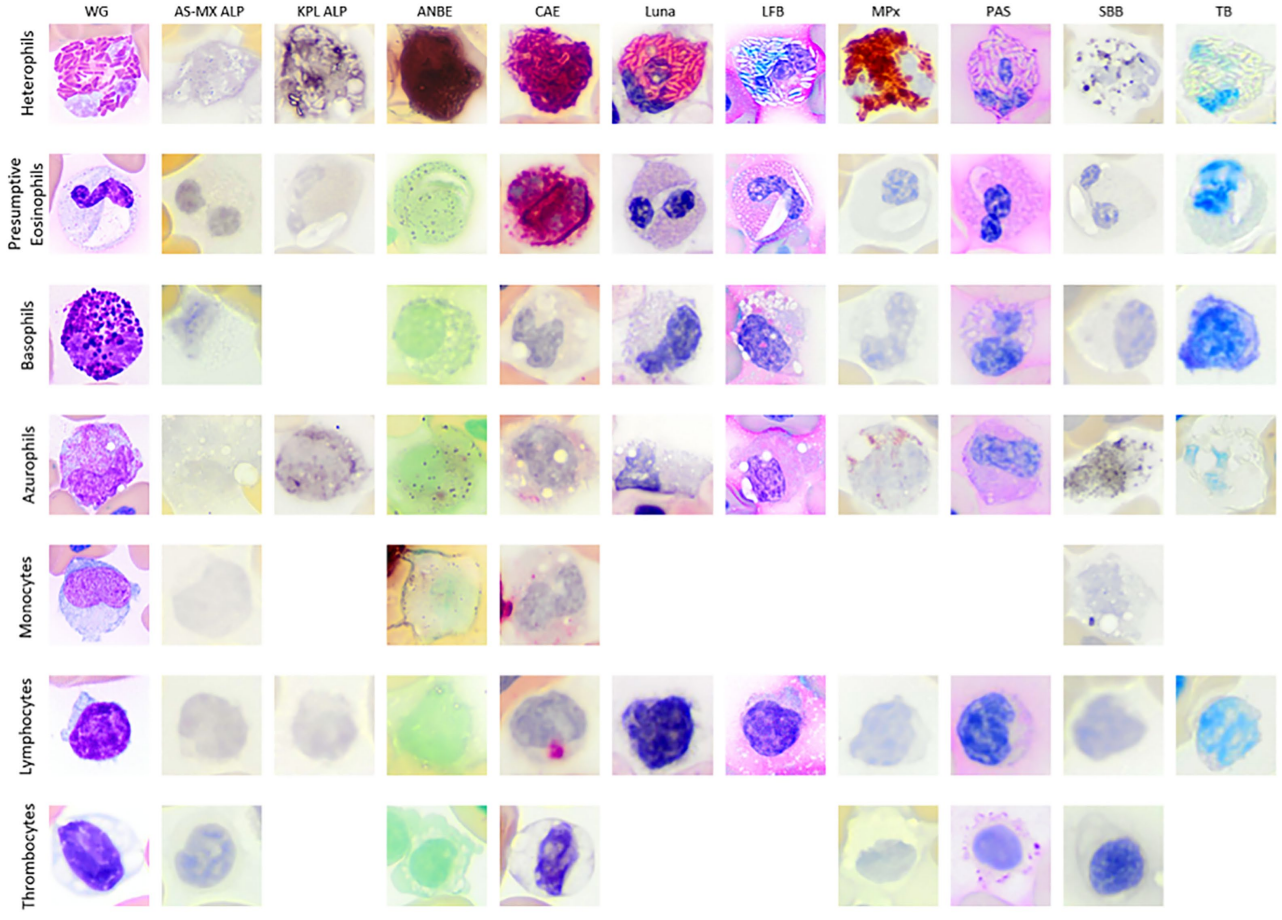
Image composite of white blood cells (WBCs) and thrombocytes of the Argentine black and white tegu (*Salvator merianae*). The WBCs are from tegu 1, except for AS-MX ALP basophil which is from tegu 2, with the blood films stained with Wright-Giemsa (WG; Romanowsky-based stain) and nine cytochemical stains (ALP, alkaline phosphatase with different substrates [AS-MX/fast blue RR and KPL BCIP/NT]; ANBE, α-naphthyl butyrate esterase; CAE, chloroacetate esterase; Luna; LFB, luxol fast blue; MPx, myeloperoxidase; PAS, Periodic acid-Schiff; SBB, Sudan black B; TB, toluidine blue). Basophils were very difficult to identify with either ALP stain and were presumed negative. Azurophils and monocytes were difficult to differentiate. Monocytes were not conclusively identified with the KPL ALP and LFB. The thrombocytes are from tegu 2 with the blood film stained with modified Wright’s stain (Romanowsky-based stain) and the same cytochemical stains except for KPL ALP, Luna, LFB, and TB, which were not performed. All images were cropped after being imaged at 100x magnification.

Heterophils from both tegus showed diffuse cytoplasmic staining with ANBE and CAE and diffuse granule uptake with MPx and SBB. Heterophil granules in tegu 1 were positive for Luna and LFB, but there was no reaction with TB (Figure 2; Table 1). There were between-tegu differences in cytoplasmic staining with PAS (positive versus variable uptake in tegu 1 and 2, respectively) and AS-MX ALP (variable versus negative uptake in tegu 1 and 2, respectively) (Figure 2; Table 1; Supplementary Figures S1A,B). The heterophil cytoplasm was stained differently with two ALP stains in tegu 1: variable weak positive stippling with AS-MX ALP and diffuse positivity with KLP ALP (Figure 2; Table 1).

The ultrastructural features of heterophils in tegu 1 were similar to those previously described (6). The granules on light microscopy corresponded with large oval organelles (up to 0.29 by 0.117 μm) on TEM. These numerous organelles contained a homogeneous electron-dense matrix lined by a single bilipid membrane. Lower numbers of small (up to 0.084 by 0.027 μm), elongated organelles containing a fine granular electron-dense matrix lined by a single bilipid membrane, were consistent with azurophilic, or primary, granules, as described in humans (16) (Supplementary Figures S2A,E). Additional organelles, such as mitochondria, rough endoplasmic reticulum (RER), and Golgi, as well as lipid droplets, were also observed. Phagosomes were apparent in a few heterophils.

### Eosinophils

The unique WBC type with cytoplasmic inclusions in tegu 1 resembled cells identified as eosinophils in tegu 2, except the latter cells lacked a visible inclusion. The cells measured ~12.5–14.5 μm and had a moderate-to-abundant amount of cytoplasm with small light blue granules. Granules were darker, larger, and more discernible in tegu 2. Most of the cells in tegu 1 had a single distinct, elongate, crescent- or ring-shaped pale, eccentrically to centrally placed inclusion (Figures 1A-D,F-P). Rare cells had no visible inclusion but similar cytoplasmic characteristics (Figure 1E). Eosinophils had segmented or bilobed nuclei with clumped chromatin.

On cytochemical staining, the cells were positive for CAE (strong granule staining) and PAS (weak diffuse cytoplasmic staining) and negative for MPx and SBB. Eosinophils were negative with both ALP stains in tegu 1 (Figure 2), whereas eosinophils in tegu 2 showed stippled cytoplasmic staining with the ALP AS-MX stain (Table 1; Supplementary Figures S1C,D). A few eosinophil granules exhibited weak focal stain uptake with LFB in tegu 1, but the cells were negative for Luna and TB. The inclusions in tegu 1 eosinophils only took up the CAE stain but were negative for the other stains (Figure 2; Table 1).

**Figure 3:**
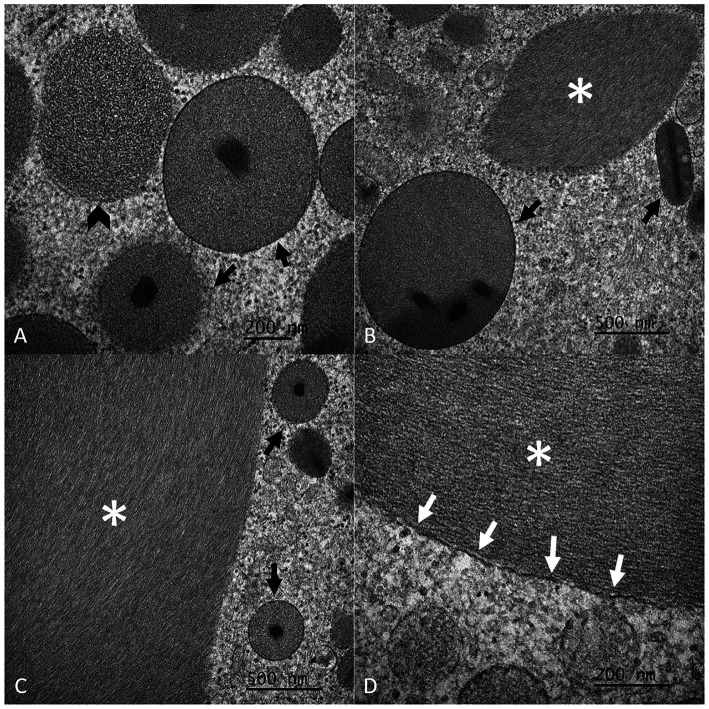
Detailed composite of the ultrastructural features of eosinophil granules and inclusions of Argentine black and white tegu (*Salvator merianae*), tegu 1, observed by transmission electron microscopy (TEM). Note the single bilipid membrane-bound granules with the round- to rod-shaped electron-dense core (crystalloid) (black arrows, **A–C**) and the non-membrane bound inclusion of moderate electron density (white asterisk, **B–D**). The granules have a granular to rarely more filamentous (black arrowhead, **A**) texture. The inclusion has a filamentous texture. Rarely, portions of the inclusion appear to be associated with a single bilipid membrane (white arrows, **D**).

Ultrastructurally, eosinophil nuclei had an ~1:1 mixture of eu- and hetero-chromatin. The cytoplasm contained many variably sized (up to 0.86 μm) round-to-oval organelles (specific granules) lined by a single bilipid membrane and containing a moderate electron-dense matrix with a granular to filamentous texture and a round- to rod-shaped electron-dense core (crystalloid). Additionally, there was a large (up to 6.31 by 2.21 μm), irregularly elongated, non-membrane-bound inclusion of moderate electron density with a filamentous texture that occasionally displayed a whirling pattern (Figure 3; Supplementary Figures S2B,F). Occasionally, segments of these inclusions appeared to be lined by a single bilipid membrane (Figure 3C). Mitochondria and RER were also observed. The TEM features of these organelles were typical of eosinophils in mammalian species.

### Basophils

On Romanowsky-based stain, basophils measured ~11 μm and had a moderate amount of colorless to lightly basophilic cytoplasm that often contained numerous small-to-large medium-to-dark purple granules. The cytoplasm was frequently obscured in cells packed with granules but ranged from colorless to light blue. A few basophils lacked granulation or contained only a few dark purple granules. The eccentric nuclei were ovoid to reniform to bilobed with clumped chromatin.

The cells were difficult to conclusively identify in both ALP stains in tegu 1, suggesting they were negative. Basophils in both tegus were negative for ANBE, CAE, MPx, and SBB (Figure 2; Table 1). In tegu 2, the cells were also negative for AS-MX ALP (Figure 2; Table 1). Basophils from tegu 1 showed variable granule stain uptake with TB but were negative for Luna and LFB (Figure 2; Table 1). Differences between tegu staining reactions were seen with PAS; tegu 1 basophils were variably positive, with weak cytoplasmic staining (Figure 2; Table 1), whereas basophils in tegu 2 showed moderate-to-strong granule uptake (Table 1).

Basophil appearance on TEM was similar to that previously described (Supplementary Figures S2C,G) (6). Their granules on light microscopy consisted of up to 1.04 μm rounded organelles lined by a single bilipid membrane containing a matrix of variable electron density and texture and various organelles, including mitochondria, RER, and Golgi.

### Azurophils

Azurophils, measuring ~12–16 μm, had a moderate amount of medium basophilic cytoplasm with fine, coalescing round- to rod-shaped purple granules and variable numbers of small clear vacuoles on Wright-Giemsa stain. Nuclei were eccentrically located and round-to-lobulated with clumped chromatin. Rare azurophils from tegu 1 contained phagocytosed heterophil granules, suggestive of activation, possibly secondary to delayed sample processing (2 h) or inflammation.

Azurophils from both tegus showed mild-to-moderate diffuse cytoplasmic staining with PAS, granular staining of differing intensity with CAE, and were variably positive for ANBE and SBB, with weak diffuse and granular cytoplasmic uptake, respectively. Azurophils from tegu 1 displayed positive cytoplasmic reactions with both ALP stains, albeit to variable degrees, and were variably positive for LFB but negative for Luna and TB (Figure 2; Table 1; Supplementary Figure S1D). Between-tegu differences were noted in azurophils for AS-MX ALP (Supplementary Figures S1E,F) and MPx (Supplementary Figures S1G,H), with positive reactions in tegu 1 but not tegu 2 (Table 1).

TEM features were similar to those previously described for azurophils (6). Azurophilic granules were composed of round-to-slightly oval organelles measuring up to 0.35 μm and consisting of a homogeneous electron-dense matrix lined by a single bilipid membrane. These features are similar to lysosomes of mammalian macrophages (17). Azurophils also contained organelles, such as mitochondria, RER, and Golgi, along with occasional phagolysosomes (Supplementary Figures S2D,H).

### Monocytes

Monocytes were not conclusively identified in tegu 2. In tegu 1, they were similar in size to azurophils and displayed features observed in other species, including light basophilic cytoplasm with occasional vacuoles and reniform to slightly lobulated eccentric nuclei. Unlike azurophils, they lacked cytoplasmic granules. Rare monocytes with phagocytosed heterophil granules were identified. As mentioned for azurophils, the phagocytosis could be due to activation.

Monocytes were difficult to differentiate from azurophils on cytochemical staining, except for AS-MX ALP (positive in azurophils and negative in monocytes). These cells were not conclusively identified in the KPL ALP and LFB stains. When observed, they had similar cytochemical staining characteristics to azurophils (Figure 2; Table 1). The ultrastructural appearance of monocytes was similar to azurophils, except they contained fewer lysosome-like organelles mentioned above and more phagolysosomes (Supplementary Figures S2I,M).

### Lymphocytes

With Romanowsky-based stain, lymphocytes were similar to those of other species and were mostly small, measuring ~6–7 μm, with scant light-to-medium blue cytoplasm, occasional blebbing borders, and round-to-ovoid eccentric nuclei with clumped chromatin. Rare granular and large reactive lymphocytes with deep basophilic cytoplasm were observed.

On cytochemical staining, a few lymphocytes were positive for CAE, exhibiting weak-to-moderate focal staining in the cytoplasm. They were negative for all other stains (Figure 2; Table 1). The ultrastructural features of lymphocytes were similar to that previously described (6); they had a small amount of cytoplasm that contained a few organelles, such as mitochondria and a Golgi apparatus (Supplementary Figures S2J,N). Low numbers of plasmacytoid lymphocytes were observed ultrastructurally, although they were not identified on a Wright-Giemsa blood film review. These cells contained an extensive amount of RER, Golgi, and mitochondria (Supplementary Figures S2K,O).

### Thrombocytes

Thrombocytes were mostly clumped and not present, or easy to identify, in all blood films from tegu 1 but were seen mostly as individualized cells in tegu 2. Individual thrombocytes measured ~6–8 μm and had a scant amount of clear-to-faint blue cytoplasm and ovoid nuclei containing condensed chromatin, which is similar to that previously reported in Romanowsky-based stain (6).

Thrombocytes were only positive for PAS in both tegus (Figure 2; Table 1), with staining on the edges of the cytoplasm in the individual cells in tegu 2. Thrombocytes from tegu 1 on TEM were similar to lymphocytes in size, nuclear shape, and the euchromatin-to-heterochromatin ratio. They did, however, contain canalicular and microtubular systems, which were slightly obscured by phagocytic activity, the latter of which is likely secondary to delayed sample processing (2 h) or activation from inflammation (Supplementary Figures S2L,P).

### Erythrocytes

Erythrocytes appeared similar to those observed in other reptilian species. Mature elliptical cells measured ~15–20 μm long and ~ 8–10.5 μm wide with red cytoplasm, which frequently contained a single pinpoint greenish-blue inclusion. The centralized, irregularly ovoid nuclei had condensed chromatin (Supplementary Figure S3). Low numbers of polychromatophils were observed in blood films from tegu 1; these cells were slightly smaller and rounder with mildly basophilic cytoplasm and a more open chromatin pattern.

On cytochemical staining, low numbers of erythrocytes had 1–4 small faint ANBE- or weak-to-moderate CAE-positive inclusions in both tegus. A few erythrocytes in tegu 1 had positive single large cytoplasmic inclusions with Luna stain and single small PAS-positive inclusions (Supplementary Figure S4). Ultrastructurally, erythrocytes contained few mitochondria and lipid droplets, and lysosomes were found to have degradative material. Rare lamellar bodies and degrading mitochondria were also observed (Supplementary Figure S3).

## Discussion

The WBC type with unique pale staining inclusions in the first Argentine black and white tegu lizard (tegu 1) displayed different morphological, cytochemical staining, and ultrastructural characteristics to the other leukocytes in the blood, supporting that they were distinct leukocytes. The ultrastructural findings confirmed they were eosinophils due to the presence of a crystalloid core in organelles corresponding to the granules observed with light microscopy. These crystalloid cores are a characteristic feature of eosinophils in many mammalian species, birds (ducks and geese), and reptiles, including the Hawaiin green sea turtle (*Chelonia mydas*) and bobtail lizard (*Tiliqua rugosa*) (18–21). In previous reports of tegu WBCs, similar ultrastructural organelle findings were identified in a “cellular fragment” (6) and an intact eosinophil (8), although neither of these cells had inclusions.

In this study, we confirm that cytoplasmic inclusions are only found in eosinophils and not in monocytes, as previously thought (6). However, rare eosinophils in tegu 1, all eosinophils in tegu 2, and those referenced by Carvalho et al. (8) lacked these inclusions, indicating that these inclusions should not be expected in all black and white Argentine tegus. The nature and clinical relevance of the inclusions are unknown. The inclusions do not have a crystalloid appearance, and no correlation can be made between the presence of these inclusions and inflammation as similar cells, with the inclusions, have been observed in closely related species, such as healthy red tegu and Savannah monitor lizards (3). The lack of reactivity with PAS and SBB suggests they are not composed of glycogen, glycogen-containing substances, or lipids. Since the inclusions have similar CAE-staining and ultrastructural patterns to the secondary granules, we speculate that they represent consolidation or fusion of eosinophil granule contents, perhaps after intracellular degranulation (potentially representing eosinophil activation). Further studies will be needed to elucidate the contents and origin of these inclusions.

Different mammalian, amphibian, and reptilian species have unique cytochemical staining characteristics in their leukocytes. Although CAE reactivity is typically a neutrophil marker in mammals, eosinophils in other non-mammalian species, including the green sea turtle (*Chelonia mydas*), yellow-bellied slider (*Trachemys scripta scripta*), and koi (*Cyprinus rubrofuscus*) (2), have been recorded as being CAE-positive. The cytochemical reactions for MPx in heterophils and azurophils and PAS in heterophils, basophils, and thrombocytes were relatively consistent with those already published for this species (8). An explanation for the staining differences between the tegus is not apparent. However, the individual cytochemical staining reactions in the tegus were not batched together, and the observed differences could be exacerbated by batch-to-batch variation in staining. Alternatively, tegu 1 had evidence of inflammation, and this could have altered the cytochemical staining characteristics of the cells and would also explain the presence of monocytes, phagocytic activity in azurophils, and plasmacytoid lymphocytes in this animal. In addition, the blood samples from the tegus were collected using different anticoagulants. The effect of anticoagulants on cytochemical staining characteristics is unknown, and it is possible that the type of anticoagulant affects the reactions. Ideally, it would be optimal to perform cytochemical staining in additional animals in this species. We have observed differences in cytochemical staining reactions between individual animals of other species, even when blood samples are collected into the same anticoagulant and staining is performed in the same batch (unpublished findings). However, these differences, similar to those observed in the tegu, are minor and unlikely to be of clinical relevance. Our findings provide further evidence that not only do morphological and cytochemical staining properties of WBCs vary between different reptile species, but they can also be different when comparing individuals of the same species.

Luna stain, which is considered useful for the identification of eosinophils in mammals and birds, cannot be used with confidence to identify eosinophils in reptiles since both heterophils and eosinophils may stain positive (2, 22). In the current study, eosinophils of both tegus were negative, and heterophils stained positive in tegu 1. Similar conclusions have been drawn in other reptilian species, such as the painted turtle (*Chrysemys picta*) (23). Heterophils also exhibited stronger uptake of LFB, another supposed eosinophil-specific stain (2), whereas only a few eosinophil granules showed weak LFB staining. Our results indicate that neither Luna nor LFB stains can be relied on to identify eosinophils with confidence in this species. The positive reaction for TB in tegu basophils suggests that this stain could be used to identify well-granulated basophils in this species, although it is unclear if the less granulated basophil variants would be positive with this stain.

We tested two different substrates for ALP in tegu 1, with the reactions being performed on the same day. The ALP staining patterns in the tegu WBC were not consistent between the stains, unlike equine neutrophils, our positive ALP control, which had similar granular staining reactions. The affinity of reptilian leukocyte ALP for different substrates is unknown; however, there are reported differences in naphthol-based substrate affinity for calf intestinal ALP (24), and the same is likely true for leukocyte ALP in tegus, and possibly other species. These results indicate that the type of substrate used to detect ALP in reptiles matters and one substrate or commercial kit cannot be readily substituted for another or be expected to yield equivalent results.

Thrombocyte PAS positivity is consistent with previous findings in birds and reptiles (2). Similar to light microscopy evaluation during blood film review in some cases, even ultrastructural differentiation between thrombocytes and lymphocytes was difficult because thrombocyte phagocytic activity hindered visualization of the canalicular and microtubule systems, the hallmark of thrombocyte identification. In Romanowsky-based stains, a few erythrocytes in both tegus contained 1–2 greenish-blue-to-dark basophilic inclusions that have been reported in other reptile species and have historically been reported as degenerate organelles or membranes thereof (1, 25). These inclusions were difficult to definitely identify ultrastructurally in tegu 1 but may represent lipid droplets or lamellar bodies. Degenerating organelles may also explain the positive cytochemical reactions in the erythrocytes, which were largely similar in both tegus.

## Conclusion

The results of our study confirm that the unique cells with or without crescent-shaped inclusions observed in some Argentine black and white tegus are eosinophils and should be classified as such during the WBC differential count. Cytochemical staining and TEM information on all WBC types in this species helped to further understand and characterize WBC types, assisted in WBC differential counting, and will likely be helpful with cell identification when extrapolated to other lizard species.

## Data availability statement

The original contributions presented in the study are included in the article/Supplementary material, further inquiries can be directed to the corresponding author.

## Ethics statement

The requirement of ethical approval was waived by University of Florida Institutional Animal Care and Use Committee for the studies involving animals because tissue protocol (No sample collection specifically for this research). The studies were conducted in accordance with the local legislation and institutional requirements.

## Author contributions

SB: Conceptualization, Investigation, Methodology, Visualization, Writing – original draft, Data curation, Formal analysis, Software, Validation. NS: Conceptualization, Funding acquisition, Investigation, Methodology, Project administration, Resources, Supervision, Visualization, Writing – original draft. AA: Investigation, Methodology, Software, Visualization, Writing – original draft. CH: Conceptualization, Investigation, Validation, Writing – review & editing. RM: Writing – review & editing. DH: Writing – review & editing. TS: Conceptualization, Data curation, Formal analysis, Investigation, Methodology, Resources, Software, Validation, Visualization, Writing – original draft.
