# Supplementary material for: A mystery revealed: an update on eosinophil and other blood cell morphology of the Argentine black and white tegu (Salvator merianae)
Source: Front Vet Sci. 2024 Jun 13;11:1387178. doi: 10.3389/fvets.2024.1387178 (PMC11208677; doi:10.3389/fvets.2024.1387178)
Supplement: Supplementary file 1 [file Data_Sheet_1.pdf]

Supplementary Material

1 Supplementary Figures

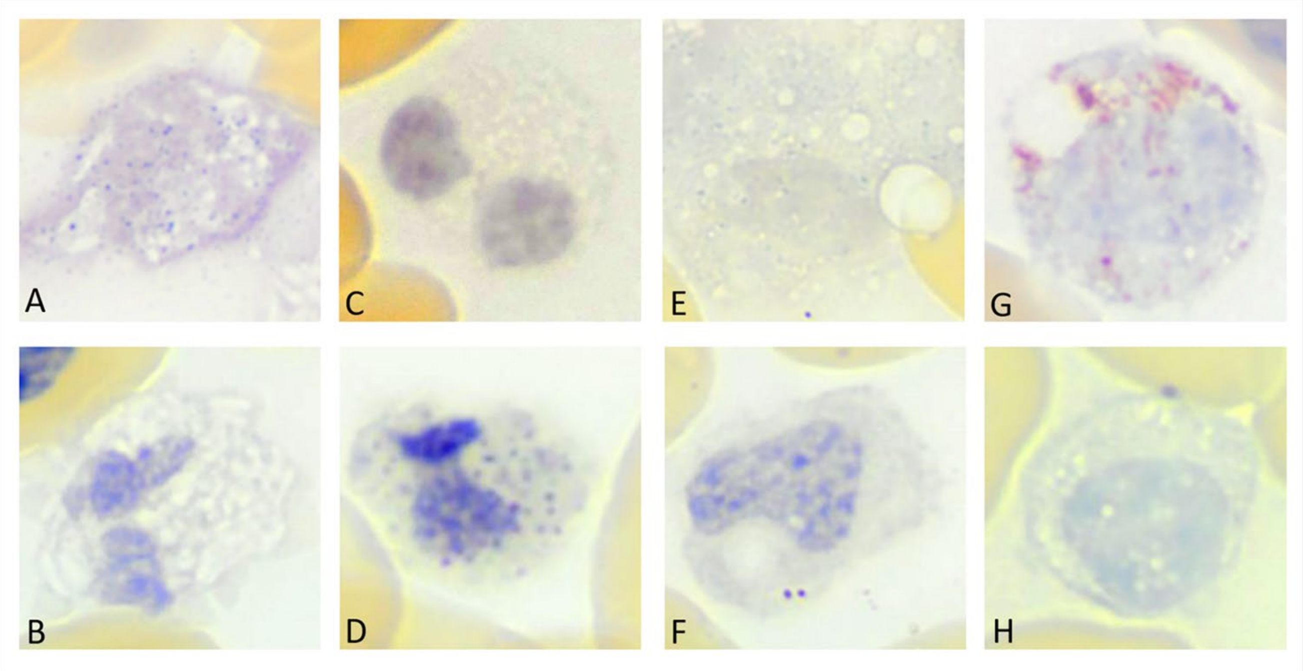

Figure S1: Image composite of white blood cells (WBCs) of the Argentine black and white tegu (Salvator merianae) showing cytochemical staining differences between tegu 1 (top row) and tegu 2 (bottom row). **(A, B)** Heterophils from tegu 1, when positive, had weak fine cytoplasmic stippling with the AS-MX ALP stain, whereas heterophils from tegu 2 were negative. **(C, D)** Eosinophils from tegu 1 were negative while eosinophils from tegu 2 had coarse cytoplasmic staining with AS-MX ALP. **(E, F)** Azurophils from tegu 1 showed weak fine cytoplasmic stippling with AS-MX-ALP, in contrast to azurophils from tegu 2, which were negative. **(G, H)** Azurophils from tegu 1 expressed multifocal granule stain uptake with MPx, whereas those from tegu 2 were negative. AS-MX ALP: AS-MX/fast blue RR Alkaline Phosphatase; MPx: Myeloperoxidase

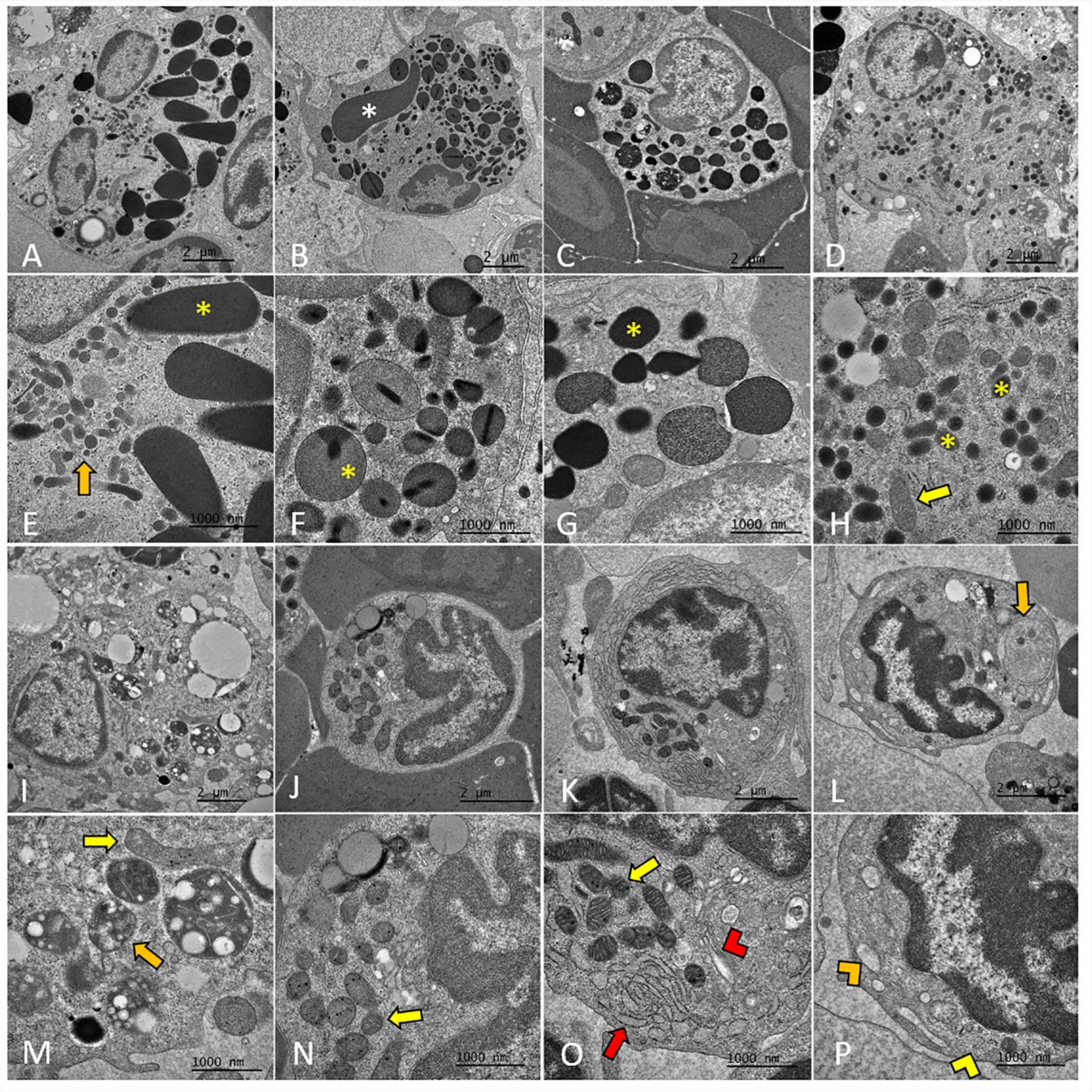

Figure S2: Composite of the ultrastructural features of Argentine black and white tegu (*Salvator merianae*), tegu 1, white blood cells (WBC) observed by transmission electron microscopy (TEM). For each WBC, the entire cell is shown in the top photomicrograph and the cytoplasmic contents are highlighted in the corresponding lower photomicrographs. Note that the term granules, as they are often described using light microscopy, is being used to describe small organelles that are outlined by a single bilipid membrane. (**A, E)** Heterophils had a lobulated nucleus and abundant cytoplasm with numerous large, dense granules (yellow asterisk) and fewer azurophilic, primary, granules (orange arrow). (**B, F)** Unique leukocytes (eosinophils) had a bilobed, segmented, or banded nucleus and abundant cytoplasm with specific granules containing a round to rod-shaped electron-dense core

(crystalloid) (yellow asterisk) along with a large, irregularly elongate, non-membrane bound inclusion of moderate electron density (white asterisk). **(C, G)** Basophils had a round or bilobed nucleus and abundant cytoplasm with many electron-dense granules (yellow asterisk). **(D, H)** Azurophils had a lower nuclear-to-cytoplasmic ratio compared to lymphocytes, a round to indented nucleus, and cytoplasm containing many lysosome-like organelles (yellow asterisk) and additional organelles, like mitochondria (yellow arrow). **(I, M)** Monocytes appeared similar to azurophils but contained more phagolysosomes (orange arrow) compared to azurophils. A mitochondrion is visible (yellow arrow). **(J, N)** Lymphocytes had a high nuclear-to-cytoplasmic ratio and a thin rim of cytoplasm with a scarcity of organelles, including mitochondria (yellow arrow) and Golgi. **(K, O)** Plasmacytoid lymphocytes also had a high nuclear-to-cytoplasmic ratio and a small amount of cytoplasm but contained extensive rough endoplasmic reticulum (red arrow) and Golgi (red arrowhead) and fewer mitochondria (yellow arrow). **(L, P)** Thrombocytes had a high nuclear-to-cytoplasmic ratio, a round nucleus, and a small amount of cytoplasm containing phagocytosed debris (orange arrow) as well as canalicular (orange arrowhead) and microtubular (yellow arrowhead) systems.

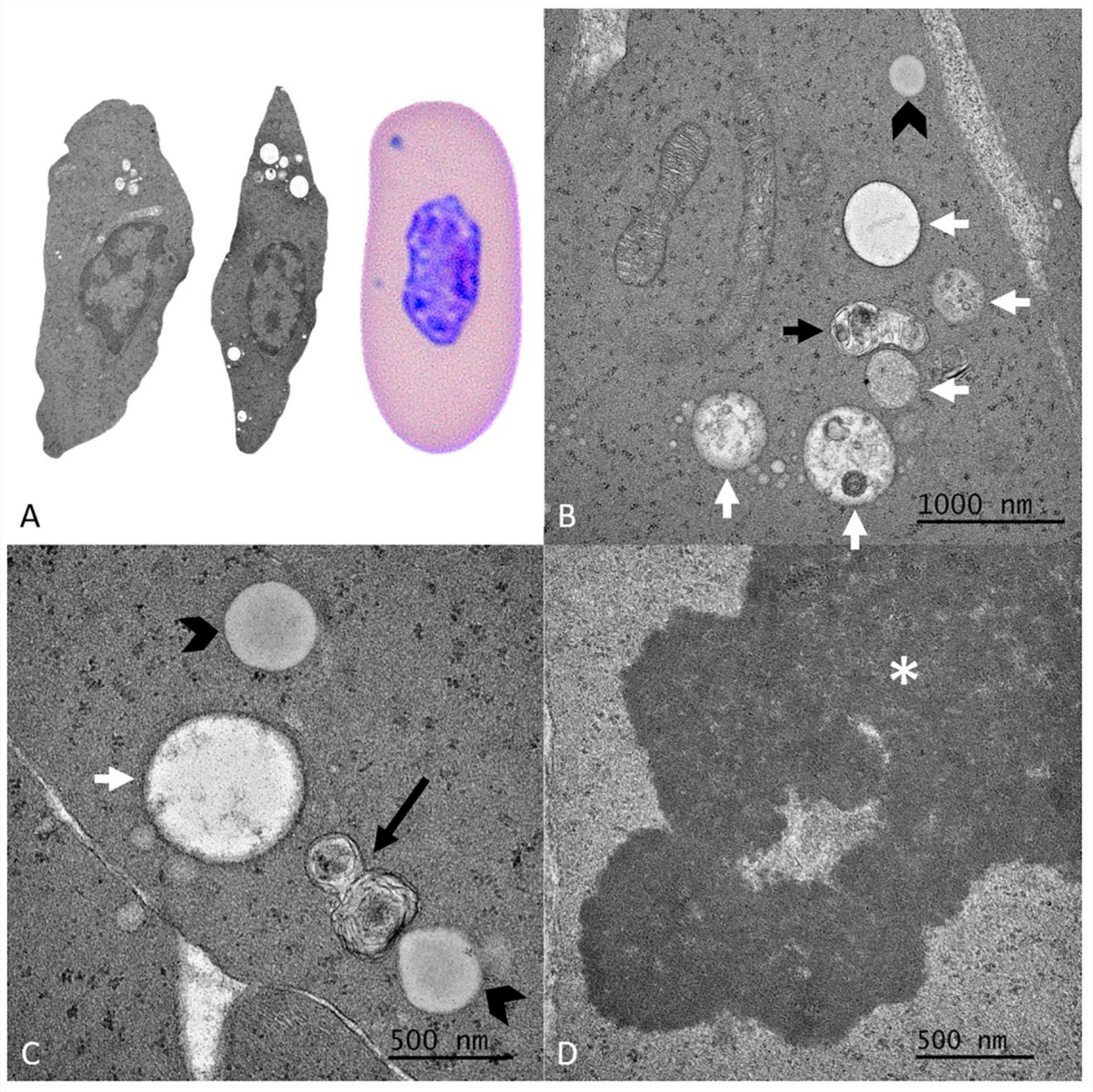

Figure S3: Composite of the ultrastructural features of Argentine black and white tegu (*Salvator merianae*), tegu 1, erythrocytes observed by transmission electron microscopy (TEM) with a Wright-Giemsa 100x inset. **(A)** Due to sectioning for TEM, erythrocytes are irregularly shaped and appear to contain vacuoles and occasional mitochondria. Two pinpoint, blue-green inclusions are observed on the Wright-Giemsa inset. **(B)** Few long, thin mitochondria, and one degrading mitochondria (center, short black arrow) are next to five membrane-bound vesicles, consistent with secondary lysosomes (white arrows), which contain variable amounts of degrading debris; small vesicles appear to be fusing with the lysosomes. One round lipid droplet (black arrowhead), of moderate electron density, is also present. **(C)** Two lipid droplets (black arrowheads) and two secondary lysosomes, one being an electron-lucent large vesicle (white arrow) closely associated with two small pinocytotic vesicles and the second being a lamellar body (long black arrow). **(D)** The large, amorphous collection of electron-dense material (white asterisk) in the cytoplasm of one erythrocyte is of unclear origin or clinical significance. A light microscopic correlate to this inclusion was not identified in Wright-Giemsa-stained blood films.

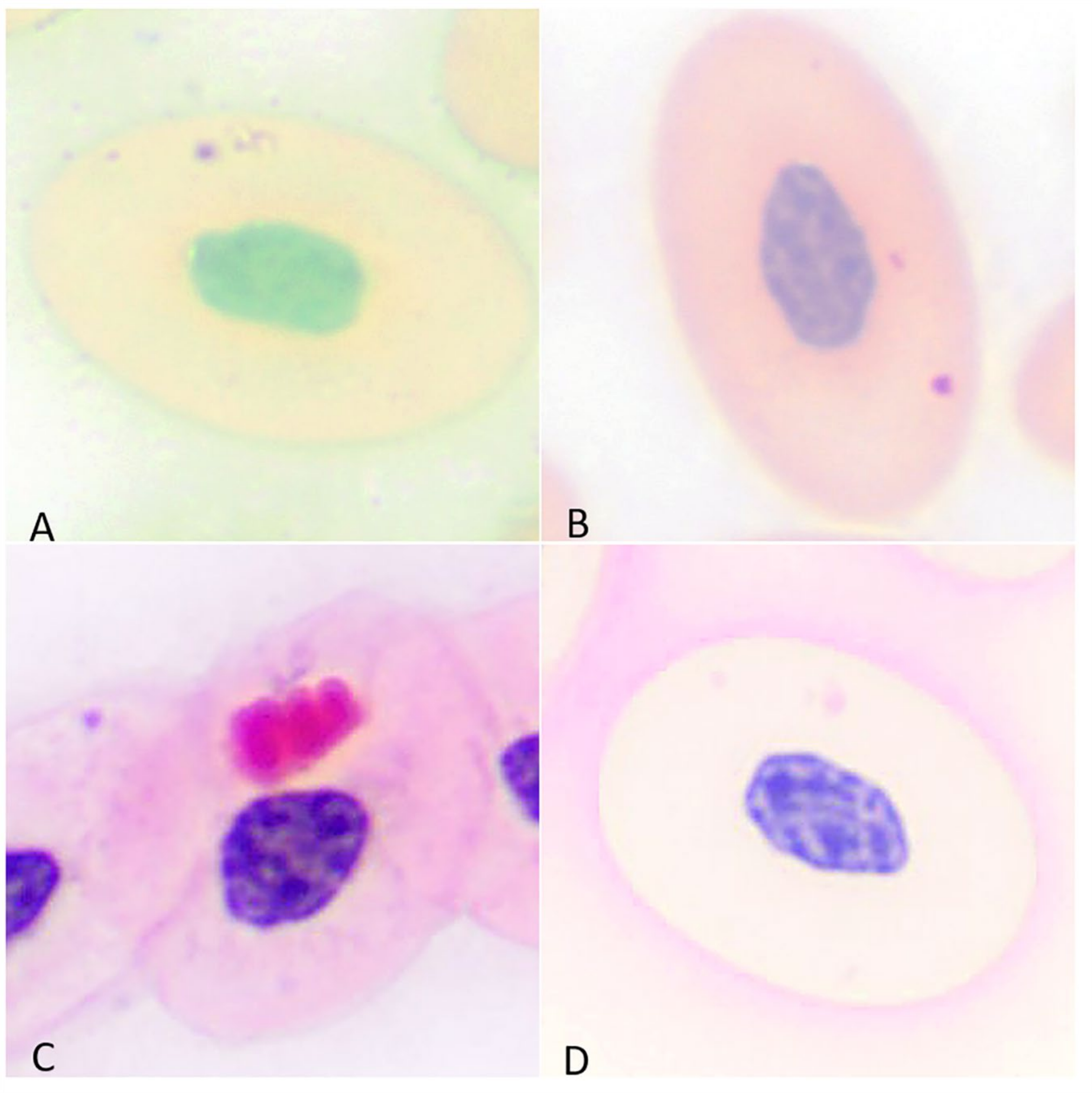

Figure S4: Image composite of erythrocytes of the Argentine black and white tegu (*Salvator merianae*) from tegu 1 stained with four different cytochemical stains. **(A)** ANBE, α-naphthyl butyrate esterase. **(B)** CAE, chloroacetate esterase. **(C)** Luna. **(D)** PAS, periodic acid-Schiff. All images were cropped after being imaged at 100x magnification.

2 Supplementary Tables

Table S1: Hematological data from two Argentine black and white tegus (*Salvator merianae*). The PCV was determined with centrifugation of a microhematocrit tube, plasma total protein was measured by refractometry, and the WBC differential leukocyte count was obtained by manual blood film review by two authors (SB tegu 1, TS tegu 2).

Test		Tegu 1	Tegu 2
Anticoagulant		Lithium heparin	EDTA
WBC	K/µL	10.2 (estimate)	8.3 (hemocytometer)
PCV	%	33	38
TP (refractometer)	g/dL	7.1	7.3
Heterophils	%	62	31
	K/µL	6.3	2.6
Immature heterophils	%	4	0
	K/µL	0.4	0
Eosinophils (in tegu 1, initially classified as “other” WBC or “unique WBC with crescentic inclusions”)	%	6	8
	K/µL	0.6	0.7
Basophils	%	11	6
	K/µL	1.1	0.5
Azurophils	%	5	14
	K/µL	0.5	1.2
Monocytes	%	2	0
	K/µL	0.2	0
Lymphocytes	%	10	40
	K/µL	1.0	3.3
Thrombocyte Estimate		Adequate; frequently clumped	Adequate; lightly clumped

WBC: white blood cell; PCV: packed cell volume; TP: total protein.
